# Is Time-Driven Activity-Based Costing Coming out on Top? A Comparison with Activity-Based Costing in the Health Field

**DOI:** 10.3390/healthcare9091113

**Published:** 2021-08-27

**Authors:** Angels Niñerola, Ana-Beatriz Hernández-Lara, Maria-Victoria Sánchez-Rebull

**Affiliations:** Department of Business Management, Faculty of Business and Economics, University Rovira i Virgili, 43204 Reus, Spain; anabeatriz.hernandez@urv.cat (A.-B.H.-L.); mariavictoria.sanchez@urv.cat (M.-V.S.-R.)

**Keywords:** time-driven activity-based costing, activity-based costing, health, research impact

## Abstract

The cost of health is a recurrent topic that has generated much research, as it affects all of society. Both public and private agents need to know the real cost of treatments, services, and products for decision-making. This article aims to compare the use and research impact of two cost systems widely used in health: ABC and TDABC, which is an evolution of ABC. For doing so, a bibliometric review in Scopus and Medline was carried out encompassing the years 2009–2019. The results show a great increase in publications using TDABC, while publications on ABC stabilized. On the other hand, the TDABC articles presented higher research impacts in traditional and alternative metrics. Articles on TDABC are more frequently cited, published in better journals, and more visible in academic social networks. The findings suggest that scholars and practitioners should focus on TDABC rather than ABC for addressing cost in health for its simplicity, projection, and research opportunities.

## 1. Introduction

There has always been a great interest in knowing the cost of treatments, services or products in the health field. There is a very extensive body of literature in this regard [1,2,3,4]. Different cost systems have been used for cost control and resource allocation improvement. The methods of activity-based costing (ABC) and, recently, time-driven activity-based costing (TDABC) represent the best alternatives to traditional systems in complex environments like health care services [1,5].

ABC was created for obtaining a more accurate cost of products [6,7]. The most significant difference between ABC and traditional systems is the allocation of indirect costs. Their assignment is based on the activities required to obtain the product or service. Therefore, ABC focuses on measuring the cost and performance of the activities which consume the resources [6]. However, it is not exempt from criticism [8]. Some critiques are related to its methodology, as defining activities is not a simple task [9,10]. Others are focused on the difficulty of its implementation [11]. Consequently, not all companies are encouraged to use it [8].

To convince skeptics and reduce criticism of the method, Kaplan and Anderson proposed the evolution of ABC with the goal of increasing simplicity and usefulness [12]. They developed TDABC to focus on time as a cost generator, thereby simplifying operations. Consequently, in recent years, this cost system has gained interest and has been widely implemented in the health field [13,14].

This paper aims to compare the health literature addressing cost issues using these two cost systems in terms of the number of publications and research impact. We seek to identify and compare the response given by research to the use of ABC and TDABC in terms of number of publications and their impact. In today’s environment, the impact of publications is crucial for their scope and visibility, as well as for obtaining new funding. There are many reasons for justifying necessity to obtain better evaluations [15,16]. For this reason, we believe that this study can shed some light on the best cost system to obtain a more significant impact and therefore achieve the objectives of academics and practitioners.

On the other hand, the assessment of research impact is not trivial. Research impact does not have a clear and single definition. Penfield et al. [15] found that understanding of the term impact differs between users and audiences. DiBartola and Hinchcliff [17] remark that three different methods for evaluating scientific literature are citation metrics, usage metrics, and alternative metrics (so-called altmetrics).

Traditionally, citations received by an article have been a classic bibliometric method to evaluate the impact of a publication [18,19]. Similarly, and also among classic bibliometric methods, high-rated or leading journals have been used as an indicator of the quality of the papers published in them. The importance or value of an article could be considered depending on the journal in which it is published [20], suggesting that higher quartile journals will publish articles with more impact. Therefore, journal impact factors are also recognized and used as an impact metric [21].

However, other indicators are emerging as an interesting option for assessing the societal impact of research and offering new ways to measure public engagement with research output [22] beyond academic impact. Altmetrics represent an alternative to traditional bibliometric measures. They are often derived from social media sites such as Twitter or Facebook, or references managers like Cited U Like or Mendeley. In this sense, the impact is measured as the number of shares, readers or likes, for example. Although altmetrics have their detractors, who state that almetrics are easier to manipulate [23] and are not standard [24] compared to traditional bibliometrics, they also offer many opportunities. In previous research, the diversity of sources and filtering, speed of dissemination, increased audiences, openness, and the different ways to track the scientific communication activity are mentioned as strengths of altmetrics [25,26].

A set of indicators sometimes confused with altmetrics is usage metrics [27]. Usage metrics target downloads and views, which are the most usual proxies for usage [28]. Although some research has attempted to relate them to traditional research impact, results are unclear [17,29,30,31].

Hence, there are multiple variables to consider when assessing the impact of an article. Our research aims to analyze, using several metrics, whether TDABC achieves more interest and impact in the health literature than its precursor, ABC. To answer this question, articles were retrieved from two well-known databases; their impact data was also retrieved.

We believe that this information will help academics and practitioners consider the best method for achieving their objectives and to know the impact their research may have when choosing one or the other.

Section 2 includes a brief literature review on ABC and TDABC in health. After explaining the methodology in Section 3, results are presented in Section 4, and finally, discussion and conclusions are summarized in Section 5 and Section 6, respectively.

## 2. Literature Review

Health care’s biggest problem is not insurance or politics [32]. Kaplan and Porter state that we measure the wrong things in the wrong way [32]. In terms of health, more expensive care is not necessarily synonymous with better care. People are most prone to pay for value rather than for volume [33]. In this sense, value is defined as the benefit achieved for the money spent [34]), and it can only be determined by considering their associated costs. In this environment, the measurement of cost is fundamental for quantifying the services provided by healthcare. ABC and TDABC are two of the cost systems most frequently used in the field [13].

TDABC brings some new concepts compared to the ABC model. While one is the evolution of the other, the fundamental difference between the two systems is the number of cost drivers used. TDABC only uses time as a cost driver; as a result, it demands fewer resources. TDABC requires only two key parameters: the capacity cost rate (CCR) and the time necessary to perform the activities. CCR is the cost of capacity-supplying resources divided by those resources’ practical capacity [35]. In this system, time equations are used to capture the complexity of the activities.

There are not many previous reviews that address the cost of healthcare with these methods, despite these methods being widely used. Previous research highlights that the application of ABC in health is restricted by areas or departments [36]. In particular, oncology and radiology departments have paid more attention than other areas to both cost systems. They represent a great bulk of research [1,37,38]. Perhaps cost-effectiveness in these areas is more critical, as they are departments with high costs associated, and for this reason, they have more implementations.

Similarly, most of the reviews carried out in the field have this departmental focus [36]. They are centred on a specific health area, such as surgery [39], oncology [1], obstetrics [40], or orthopaedics [41,42]. These reviews mainly focus on how the different methods (including ABC or TDABC) have measured a specific aspect within the study area [1], the variations in the use of the cost method itself, with the conclusion that it lacks standardization [42], or the comparison of profitability between different techniques [40].

The few general reviews on ABC and TDABC that were found [2,36,43] made a descriptive analysis of the literature without comparing both methods; furthermore, neither method was compared in terms of impact. We believe that this research aspect, the impact of the publications, represents a gap in the research and therefore an interesting topic to address.

On the other hand, financial constraints on the healthcare system demand for value-based models to contain cost. In this sense, cost systems, such as ABC and TDABC, could help health institutions to improve value by reducing cost while maintaining or improving outcomes.

## 3. Methodology

### 3.1. Sample Identification

**Figure 1:**
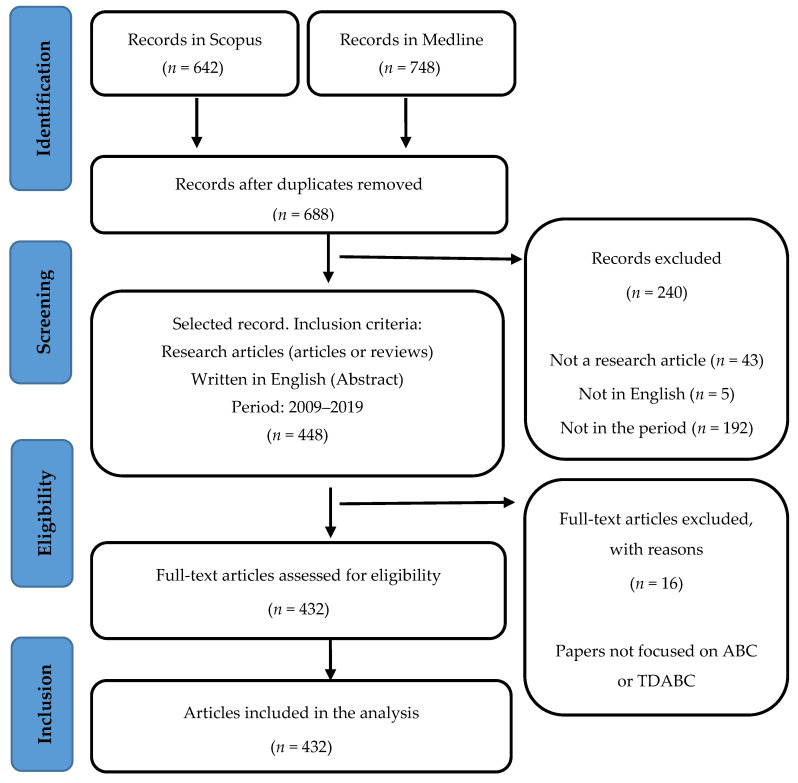
PRISMA flow adapted from Moher 2009 (search conducted on 18 September 2020).

For carrying out the bibliometric analysis, we relied on the PRISMA flow (preferred reporting items for systematic reviews and meta-analyses) for identifying papers [44] (Figure 1). In this way, we expose in detail the steps to reach the final sample included in the analysis. We considered articles published in peer-reviewed journals included in Medline or Scopus. They are popular databases in health science and management. The search included keywords such as: ‘activity-based costing’ and “time-driven activity-based costing”, as they are the most representative of these cost systems. Additionally, we included their variations: capital letters, lowercase, and removing hyphens between words. These words had to appear in the title, abstract or keywords in Scopus or in the topic in Medline.

On the other hand, we started the search in 2009, as we did not find papers using TDABC applied in the health field before this year. Including previous years would therefore include only ABC, which would skew the comparison. 

Statistical data analysis was conducted using RStudio (version 1.4.1103) (RStudio, Inc., Boston, MA, USA) [45].

### 3.2. Variables Analyzed

As mentioned before, research impact can be measured from different perspectives and with several indicators. To compare the impact of the publications using ABC and TDABC as a cost system in health, we included six indicators.

On the one hand, as academic indicators, we used paper-level indicators and journal-level indicators. Regarding the impact of the paper, we considered paper citations in the databases and the FWCI (Field-Weighted Citation Impact), an article metric used in Scopus for normalizing citation impact by field [46]. It indicates how the number of citations received by a publication compares to the average or expected number of citations received by other similar publications in terms of year, type, and discipline. Therefore, a FWCI higher than 1 means that the output is more cited than expected according to the global average.

The number of citations obtained from Scopus and Medline was independently checked by two authors to verify that all were included in the sample and were not duplicated. Moreover, we divided the value by the number of years since the paper was published (Relative Citation) to diminish the time effect in the analysis, because typically, older documents tend to have more citations.

At the journal level, we used the SJR (SCImago Journal Rank) of the year of publication and the H-Index Journal, both from the SCImago webpage [47]. The SJR indicator is a measure of the scientific influence of scholarly journals that accounts for the number of citations received by a journal and the prestige of the journals where the citations come from. It should be noted that in 97.5% of the papers we could identify these indicators.

On the other hand, among the usage metrics, we chose the abstract views in the databases; regarding altmetrics, we chose Mendeley Readers, as they are common providers of altmetrics data [48]. We could identify these values in most of the papers analyzed. Twitter and other social media for researchers shed fewer results, as they are tools for communication between researchers, but are not frequently used to share information about scientific publications [49].

## 4. Results

**Figure 2:**
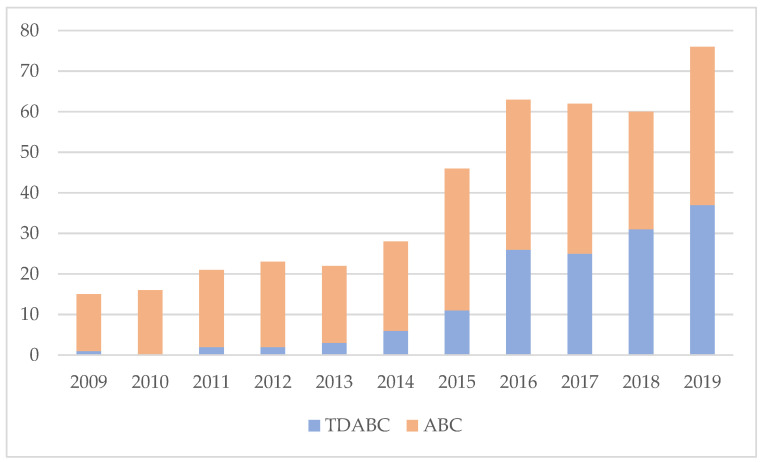
ABC and TDABC publication trends in health literature.

Four hundred thirty-two articles were included in the analysis. 288 articles use ABC for addressing cost issues and 144 use TDABC. Therefore, the ABC sample is double that of TDABC. As shown in Figure 2, the evolution of TDABC in the last four years demonstrates the equality of the two cost systems in academic publications. In fact, the growth of publications is due to the simplified cost system. It is noteworthy that in the last five years, the number publications on TDABC have increased by more than 200%. On the contrary, ABC papers remain constant, at around 35 per year.

The characteristics of the sample and descriptive statistics are detailed in Table 1 (The shaded column corresponds to ABC). Concerning citations, it can be seen that the average number of citations that TDABC papers receive per year is almost double that of ABC, with similar deviation and right-skewness, which denotes that most of the values are near zero and do not present a normal distribution. 

The behavior of the FWCI shows that the papers that use TDABC are cited more frequently than expected, since their value is more than 1. However, on average, the FWCI of the articles addressing ABC are not cited as was expected. 

The journal indicators (SJR and H Index of the journal) also show higher values for TDABC, suggesting that the journals where these papers are published are journals with higher impact, at least in the observed metrics.

On the other hand, ABC only surpasses TDABC in the number of abstract views.

Finally, all variables have the same behavior in terms of distribution: skewed to the right with the median lower than the mean and very high dispersion, especially in usage and altmetrics (abstract views and Mendeley Readers) as those variables are more unpredictable [50].

We checked the normality of the variables with Kolmogrov’s non-parametric test. We can say that none of the variables presents the normality condition to use parametric tests for the assessment the hypothesis of whether or not there is a difference in impact between the two cost systems.

Therefore, we use the Wilcoxon test (also known as Mann–Whitney test) as the non-parametric alternative to the unpaired two-samples t-test to compare ABC and TDABC samples [51]. Results in Table 2 allow us to reject the hypothesis of equality for all variables, except for abstract views (*p*-value > 0.05). Therefore, it has been demonstrated that the research impact of the papers using TDABC is statistically higher in almost all the impact metrics considered, except for usage metrics. 

## 5. Discussion

We analyzed a large sample of papers using ABC or TDABC as a cost system in some health processes or departments without focusing on a specific area. Other cost reviews paid attention to the cost of cancer [1], orthopaedics [41], or radiology [37]. Our work presented a comparison of ABC and TDABC studies applied in healthcare in general.

The findings showed a current interest in researching cost issues through ABC and even more through TDABC. The increase in papers in the healthcare field using these cost systems has also been confirmed by other researchers who have carried out literature reviews on costs in this field [2]. The need for knowing the cost of healthcare more accurately makes these cost models more usable than traditional models [43,52,53]. They are especially useful for increasing technical efficiency in the creation of healthcare products [43] On the other hand, we can say that TDABC has been responsible for the literature growth in recent years. 

Kaplan, the creator of both cost systems, has written numerous papers in the last two decades advocating for TDABC, making his preference clear [12,54]. On the contrary, Siguenza-Guzman et al. [13] argue that it should be considered a complement to the traditional ABC model rather than its replacement. 

Specifically in health, some authors have found that TDABC could provide a clearer idea of costs, help with resource allocation and waste reduction, and support clinicians and managers in increasing value more accurately and transparently [53,55]. In this sense, Ken Lee et al. [33] state that the TDABC approach is practical, valid, and scalable and can establish the foundation for measuring value. Knowing the remarkable importance of value in the field, it represents one of the best alternatives to traditional cost systems. 

During these years where the two cost systems have coexisted, we found a great interest in both; however, we believe that TDABC has more projection according to the literature analyzed. This is based, on the one hand, by its year-on-year growth rate, and on the other, for its research impact and greater visibility. 

Traditional academic indicators of success, such as citation indicators, show that using TDABC garners more mentions from others. Some authors have tried to explain why a paper receives more citations [56]. For example, author surname initials [57], article page count and the number of authors [58], if the paper is open access [18,59] or if it covers a hot topic [60] could have influence in this regard. TDABC is more recent than ABC and is considered by most authors to be an evolution of ABC, as it overcomes some of the criticisms of the ABC system. Therefore, the TDABC the topic seems more interesting for research. Moreover, TDABC has many implementations, which generates more case study works. This turns into more citations of similar works of those that have used the methodology previously. For this reason, citations are higher than in ABC.

Another interesting result is that TDABC papers are published in more high-ranked journals according to the metrics analyzed; hence, it is expected to achieve higher visibility and notoriety. Journal impact is a common metric for evaluating research [56,61]. Therefore, authors should pay special attention to publish their articles in the best possible journals according to their topic.

On the other hand, the previous literature highlights other less traditional metrics for addressing research impact. They seek to increase the impact of research in society in different ways, such as by gaining a more general audience not specific to academics, promoting research dissemination, generating discussion in forums, or sharing preliminary findings. Moreover, altmetrics are particularly promising to help researchers identify recently published articles that have attracted much attention [24]. We found that the number of TDABC readers in Mendeley is significantly higher than the number of ABC readers. This fact reinforces the argument of considering TDABC a hot topic in health management and the most successful method if the increase of the research impact is the target. 

We could not find differences between the samples compared in this study regarding abstract views. A possible reason could be that there are varied factors that explain the abstracts that researchers or readers consult, as they work as a first filter, together with the title, to select works related to specific topics. However, that does not guarantee their complete reading, use, or their mention in future research. Researchers cite only which articles they find most interesting or relevant to their work, blurring this indicator. A criticism of this indicator is that individuals can generate large numbers of views while browsing the literature online without actually reading the articles [17] or citing them later on [62].

## 6. Conclusions

This paper contributes to the health literature by showing that the evolved cost system is currently more successful than its predecessor. TDABC developed as an evolution of ABC, giving it more applicability because it simplifies the implementation process. Its research has more impact in the academic world than papers using ABC and also reaches wider and more general audiences according to the findings in the altmetrics. Moreover, consistency exists in both academic community evaluation (citation and FWCI) and public attention (altmetrics).

Therefore, our recommendation for authors is to focus their research on TDABC. Previous literature has proven that it generates positive results for cost control and cost distribution at the practitioner level. At the publication level, we believe that it offers more possibilities and reaches journals with higher impact.

Despite the contribution, the paper also has some limitations. The search was conducted in a limited number of databases. We excluded ABC as an acronym in the search since it returned a large number of papers not related to the accounting management area. ABC is also used as an acronym for multiple techniques and methods; for example, it can stand for approximate Bayesian computation, active breathing control or ATP binding cassette. On the other hand, we chose some representative indicators for each type of impact measure: article, journal and altmetrics. However, further investigations could include others and contrast the consistency of the results. These limitations can be opportunities for future research.

Future research could conduct a comprehensive systematic review to obtain an overview of the department where the methods are implemented, the objective pursued, and implementation success factors.

## Figures and Tables

**Table 1:** Sample characteristics.

Variable	Relative Citation		FWCI		SJR		H Index Journal		Abstract Views		Mendeley Readers	
	ABC	TDABC	ABC	TDABC	ABC	TDABC	ABC	TDABC	ABC	TDABC	ABC	TDABC
*n*	288	144	271	130	283	138	286	138	270	130	271	130
Mean	1.562	3.108	0.711	3.79	0.877	1.323	63.82	92.43	259.7	187.6	73.77	98.1
Median	0.889	2.00	0.410	1.09	0.682	1.070	43.00	75.50	47.0	157.6	42.00	73.0
Std. Dev	3.566	3.797	1.094	25.27	0.783	1.492	62.29	94.58	547.9	448.6	118.0	99.45
Skewness	8.901	2.312	4.265	11.18	1.925	6.182	1.821	3.89	3.83	7.302	5.495	2.336
Min	0.000	0.000	0.000	0.00	0.102	0.00	3.00	3.00	0.00	0.00	0.00	0.00
Max	44.90	20.00	9.630	289.0	5.541	14.97	331	747.0	3717	4505	1268	619.0
1st Qu	0.195	0.667	0.000	0.330	0.267	0.666	18.00	42.25	2.0	13.25	17.00	26.0
3rd Qu	1.800	4.00	0.8600	1.988	1.220	1.570	88.00	118.0	252.0	181.8	84.00	140.0

**Table 2:** Comparison of research impact using the Wilcoxon Mann–Whitney test.

	Relative Citation	FWCI	SJR	H Index Journal	Abstract Views	Mendeley Readers
W statistic	14,017	11,744	13,869	14,421	16,526	13,510
*p*-value	3.436 × 10^−8^	5.23 × 10^−8^	1.382 × 10^−6^	1.319 × 10^−5^	0.3422	0.0001581

## Data Availability

Data is available from the authors upon reasonable request.

## References

[1] Alves R.J.V., da Silva Etges A.P.B., Neto G.B., Polanczyk C.A. (2018). Activity-Based Costing and Time-Driven Activity-Based Costing for Assessing the Costs of Cancer Prevention, Diagnosis, and Treatment: A Systematic Review of the Literature. Value Health Reg. Issues.

[2] Keel G., Savage C., Rafiq M., Mazzocato P. (2017). Time-driven activity-based costing in health care: A systematic review of the literature. Health Policy.

[3] Larsen J., Skjoldborg U.S. (2004). Comparing systems for costing hospital treatments: The case of stable angina pectoris. Health Policy.

[4] West T.D., Balas A., West D.A.B.T.-H.F.M. (1996). Contrasting RCC, RVU, and ABC for managed care decisions. Healthc. Financ. Manag..

[5] Lievens Y., Van Den Bogaert W., Kesteloot K. (2003). Activity-based costing: A practical model for cost calculation in radiotherapy. Int. J. Radiat. Oncol. Biol. Phys..

[6] Cooper R., Kaplan R.S. (1988). Measure costs right: Make the right decisions. Harv. Bus. Rev..

[7] Cooper R., Kaplan R.S. (1991). Profit Priorities from Activity-based Costing. Harv. Bus. Rev..

[8] Geri N., Ronen B. (2005). Relevance lost: The rise and fall of activity-based costing. Hum. Syst. Manag..

[9] Nassar M., Al-Khadash H.A., Sangster A., Mah’D O. (2013). Factors that catalyse, facilitate and motivate the decision to implement activity-based costing in Jordanian industrial companies. J. Appl. Account. Res..

[10] Somapa S., Cools M., Dullaert W. (2012). Unlocking the potential of time-driven activity-based costing for small logistics companies. Int. J. Logist. Res. Appl..

[11] Beaulieu P., Lakra A. (2005). Coverage of Criticism of Activity-Based Costing in Canadian Textbooks. Can. Account. Perspect..

[12] Kaplan R.S., Anderson S.R. (2007). Time-Driven Activity-Based Costing: A Simpler and More Powerful Path to Higher Profits.

[13] Siguenza-Guzman L., Van den Abbeele A., Vandewalle J., Verhaaren H., Cattrysse D. (2013). Recent Evolutions in Costing Systems: A Literature Review of Time-Driven Activity-Based Costing. Rev. Bus. Econ. Lit..

[14] Areena S.N., Abu M.Y. (2019). A Review on Time-Driven Activity-Based Costing System in Varioius Sectors. J. Mod. Manuf. Syst. Technol..

[15] Penfield T., Baker M.J., Scoble R., Wykes M.C. (2014). Assessment, evaluations, and definitions of research impact: A review. Res. Eval..

[16] Wang J., Shapira P. (2015). Is There a Relationship between Research Sponsorship and Publication Impact? An Analysis of Funding Acknowledgments in Nanotechnology Papers. PLoS ONE.

[17] DiBartola S.P., Hinchcliff K.W. (2017). Metrics and the Scientific Literature: Deciding What to Read. J. Vet. Intern. Med..

[18] Antelman K. (2004). Do Open-Access Articles Have a Greater Research Impact?. Coll. Res. Libr..

[19] Garfield E. (1972). Citation analysis as a tool in journal evaluation. Science.

[20] Powdthavee N., Riyanto Y.E., Knetsch J.L. (2018). Lower-rated publications do lower academics’ judgments of publication lists: Evidence from a survey experiment of economists. J. Econ. Psychol..

[21] Thelwall M., Wilson P. (2014). Regression For Citation Data. J. Informetr..

[22] Bornmann L. (2014). Do altmetrics point to the broader impact of research? An overview of benefits and disadvantages of altmetrics. J. Informetr..

[23] Thelwall M., Haustein S., Larivière V., Sugimoto C.R. (2013). Do Altmetrics Work? Twitter and Ten Other Social Web Services. PLoS ONE.

[24] Adie E., Roe W. (2013). Altmetric: Enriching scholarly content with article-level discussion and metrics. Learn. Publ..

[25] Priem J., Taraborelli D., Groth P., Neylon C. (2010). Altmetrics: A Manifesto. https://digitalcommons.unl.edu/scholcom/185.

[26] Wouters P., Costas R. (2012). Users, Narcissism and Control—Tracking the Impact of Scholarly Publications in the 21st Century.

[27] Glänzel W., Gorraiz J. (2015). Usage metrics versus altmetrics: Confusing terminology?. Scientometrics.

[28] Gorraiz J., Gumpenberger C., Schlögl C. (2014). Usage versus citation behaviours in four subject areas. Scientometrics.

[29] Markusova V., Bogorov V., Libkind A. (2018). Usage metrics vs classical metrics: Analysis of Russia’s research output. Scientometrics.

[30] McGillivray B., Astell M. (2019). The relationship between usage and citations in an open access mega-journal. Scientometrics.

[31] Schloegl C., Gorraiz J. (2010). Comparison of citation and usage indicators: The case of oncology journals. Scientometrics.

[32] Kaplan R.S., Porter M.E. (2011). How to Solve the Cost Crisis in Health Care Understanding the Value of Health Care. Harv. Bus. Rev..

[33] Ken Lee K.H., Matthew Austin J., Pronovost P.J. (2016). Developing a Measure of Value in Health Care. Value Health.

[34] Marzorati C., Pravettoni G. (2017). Value as the key concept in the health care system: How it has influenced medical practice and clinical decision-making processes. J. Multidiscip. Healthc..

[35] Pannu T.S., Zygourakis C.C., Ames C.P., Ratliff J., Albert T.J., Cheng J., Knightly J. (2019). Concepts of Risk Stratification in Measurement and Delivery of Quality. Quality Spine Care: Healthcare Systems, Quality Reporting, and Risk Adjustment.

[36] Stefano N.M., da GraÃ M., LisbÃ P., Casarotto Filho N. (2012). Activity-Based Costing: Estado Da Arte Proposta Pelo Pesquisador E Revisão Bibliométrica Da Literatura. Iberoam. J. Proj. Manag..

[37] Choudhery S., Hanson A.L., Stellmaker J.A., Ness J., Chida L., Conners A.L. (2021). Basics of time-driven activity-based costing (TDABC) and applications in breast imaging. Br. J. Radiol..

[38] Laviana A.A., Ilg A.M., Veruttipong D., Tan H.J., Burke M.A., Niedzwiecki D.R., Kupelian P.A., King C.R., Steinberg M.L., Kundavaram C.R. (2016). Utilizing time-driven activity-based costing to understand the short- and long-term costs of treating localized, low-risk prostate cancer. Cancer.

[39] Najjar P.A., Strickland M., Kaplan R.S. (2017). Time-Driven Activity-Based Costing for Surgical Episodes. JAMA Surg..

[40] Toronto Health Economic Technology Assessment (THETA) Collaborative (2013). Hysteroscopic tubal sterilization: A health economic literature review. Ont. Health Technol. Assess. Ser..

[41] Blaschke B.L., Parikh H.R., Vang S.X., Cunningham B.P. (2020). Time-Driven Activity-Based Costing: A Better Way to Understand the Cost of Caring for Hip Fractures. Geriatr. Orthop. Surg. Rehabil..

[42] Pathak S., Snyder D., Kroshus T., Keswani A., Jayakumar P., Esposito K., Koenig K., Jevsevar D., Bozic K., Moucha C. (2019). What Are the Uses and Limitations of Time-driven Activity-based Costing in Total Joint Replacement?. Clin. Orthop. Relat. Res..

[43] Jacobs J.C., Barnett P.G. (2017). Emergent Challenges in Determining Costs for Economic Evaluations. Pharmacoeconomics.

[44] Moher D., Liberati A., Tetzlaff J., Altman D.G., Group T.P. (2009). Preferred Reporting Items for Systematic Reviews and Meta-Analyses: The PRISMA Statement. PLoS Med..

[45] RStudio Team (2015). RStudio: Integrated Development Environment for R: Boston, MA, USA. http://www.rstudio.com/.

[46] Purkayastha A., Palmaro E., Falk-Krzesinski H.J., Baas J. (2019). Comparison of two article-level, field-independent citation metrics: Field-Weighted Citation Impact (FWCI) and Relative Citation Ratio (RCR). J. Informetr..

[47] Scimago SJR Scimago Journal and Country Rank. https://www.scimagojr.com/index.php.

[48] Li X., Thelwall M., Giustini D. (2012). Validating online reference managers for scholarly impact measurement. Scientometrics.

[49] Holmberg K., Thelwall M. (2014). Disciplinary differences in Twitter scholarly communication. Scientometrics.

[50] Gumpenberger C., Glänzel W., Gorraiz J. (2016). The ecstasy and the agony of the altmetric score. Scientometrics.

[51] Sawilowsky S.S. (2005). Misconceptions leading to choosing the t test over the wilcoxon mann-whitney test for shift in location parameter. J. Mod. Appl. Stat. Methods.

[52] Alsayegh M.F. (2020). Activity Based Costing around the World: Adoption, Implementation, Outcomes and Criticism. J. Account. Financ. Emerg. Econ..

[53] Cannavacciuolo L., Illario M., Ippolito A., Ponsiglione C. (2015). An activity-based costing approach for detecting inefficiencies of healthcare processes. Bus. Process Manag. J..

[54] Kaplan R.S., Anderson S.R. (2004). Time-Driven Activity-Based Costing. Harv. Bus. Rev..

[55] da Silva Etges A.P.B., Ruschel K.B., Polanczyk C.A., Urman R.D. (2020). Advances in Value-Based Healthcare by the Application of Time-Driven Activity-Based Costing for Inpatient Management: A Systematic Review. Value Health.

[56] Mingers J., Xu F. (2010). The drivers of citations in management science journals. Eur. J. Oper. Res..

[57] Huang W. (2014). Do ABCs get more Citations than XYZs?. Econ. Inq..

[58] Ahmed A., Adam M., Ghafar N.A., Muhammad M., Ebrahim N.A. (2016). Impact of Article Page Count and Number of Authors on Citations in Disability Related Fields: A Systematic Review Article. Iran. J. Public Health.

[59] Davis P.M. (2011). Open access, readership, citations: A randomized controlled trial of scientific journal publishing. FASEB J..

[60] Xiao S., Yan J., Li C., Jin B., Xiangfeng W., Yang X., Chu S.M., Zha H. (2016). On modeling and predicting individual paper citation count over time. IJCAI Int. Jt. Conf. Artif. Intell..

[61] Seglen P.O. (1997). Why the impact factor of journals should not be used for evaluating research. BMJ.

[62] Giustini A.J., Axelrod D.M., Lucas B.P., Schroeder A.R. (2020). Association between Citations, Altmetrics, and Article Views in Pediatric Research. JAMA Netw. Open.

